# Kernohan–Woltman notch phenomenon in patient with subdural hematoma and ipsilateral hemiparesis in Bukavu

**DOI:** 10.1002/ccr3.7643

**Published:** 2023-07-04

**Authors:** Roméo Bujiriri Murhega, Maheshe Balemba Ghislain, Paterne Safari Mudekereza, Sudi Musilimu, Igega Bisimwa, Paul Munguakonkwa Budema, Léon‐Emmanuel Mubenga

**Affiliations:** ^1^ Department of Surgery Provincial General Reference Hospital of Bukavu Bukavu Democratic Republic of Congo; ^2^ Faculty of Medicine Université Catholique de Bukavu Bukavu Democratic Republic of Congo; ^3^ Department of Neurosurgery National Hospital of Niamey Niamey Niger; ^4^ Department of Radiology Provincial General Reference Hospital of Bukavu Bukavu Democratic Republic of Congo

**Keywords:** Bukavu, Kernohan–Woltman, subdural hematoma

## Abstract

**Key Clinical Message:**

Kernohan–Woltman phenomenon is a rare and paradoxical neurological situation in which a transtentorial lesion leads to compression of the contralateral cerebral peduncle responsible for compression of the descending corticospinal fibers with clinical consequence of a motor deficit ipsilateral to the primary lesion. This phenomenon should attract the attention of clinicians in order to avoid unfortunate incidents such as wrong‐side craniotomy in neurosurgical practice. In this work, we report a similar situation.

**Abstract:**

The Kernohan–Woltman notch phenomenon is a rare and paradoxical neurological situation in which transtentorial damage is observed leading to compression of the contralateral cerebral peduncle responsible for compression of descending corticospinal fibers with the clinical consequence of a motor deficit ipsilateral to the primary lesion. This phenomenon has been found in several situations including tumors and cerebral hematomas after craniocerebral trauma. In this work, we have reported the case of a 52‐year‐old man with hemiparesis ipsilateral to a large chronic subdural hematoma.

## 1 INTRODUCTION

The Kernohan–Woltman notch phenomenon is a neurological situation in which the patient presents with hemiparesis ipsilateral to a primary lesion in the setting of transtentorial involvement, where the contralateral cerebral peduncle is compressed against the free edge of the tentorium, responsible for compression of fibers of the descending corticospinal tract.^1^, ^2^

This phenomenon is a false sign of localization that can lead to confusion of topographic diagnosis in neurosurgical practice. It was first described by Kernohan and Woltman in 1929, through postmortem studies of 297 patients following cases of false localization observed at the time.^3^

The causes of this phenomenon are multiple and among them, we have slowly evolving supratentorial extraparenchymal mass lesions; such as neoplasms or chronic subdural hematomas.^4^ We report the case of a 52‐year‐old man who presented a few days after being beaten, a decreased level of consciousness, right mydriasis, and motor deficit of the right hemisphere caused by a large chronic right hemispheric subdural hematoma, which was immediately evacuated with a good clinical course.

## 2 CASE REPORT

We report the case of a 52‐year‐old man who, 1 month before his admission to our hospital, received a blow to the head in the right parietal region, then fell, the mechanism of which was not specified by those accompanying him, and presented with a brief initial loss of consciousness. One week after the fall, he began to present with holocranial headaches. Due to a lack of means to seek medical attention, he attended a prayer room with no change in symptomatology. Six hours before his admission, he presented with an abrupt alteration in consciousness, which motivated his relatives to bring him to our hospital for better management. On admission, he had a Glasgow score of 12 (O3V4M5), anisocoric pupils with reflex mydriasis to right light. He also had right central facial paralysis and right hemiparesis.

**FIGURE 1:**
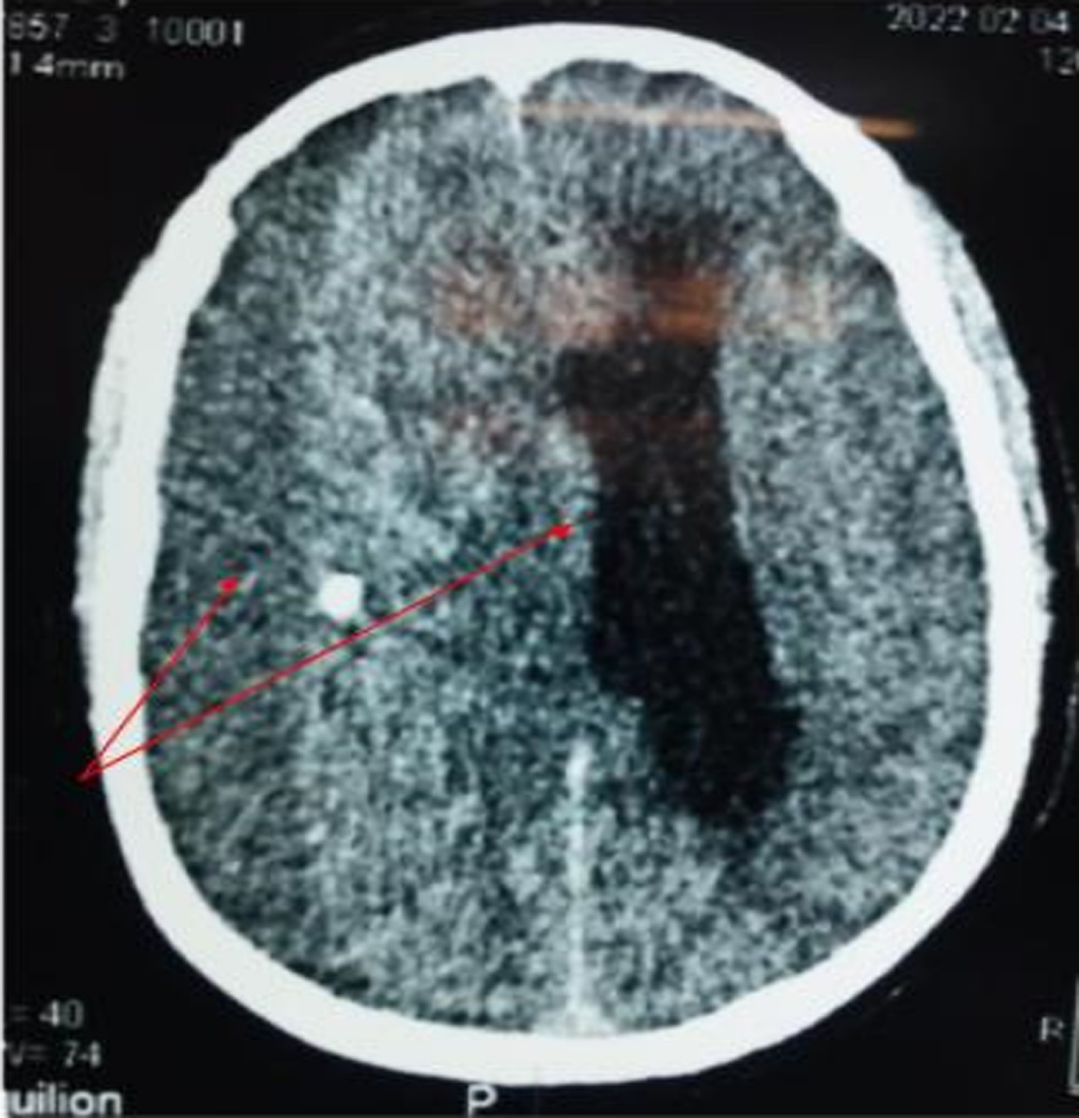
Cerebral CT image, an axial section showing a voluminous right hemispheric chronic subdural hematoma with mass effect.

**FIGURE 2:**
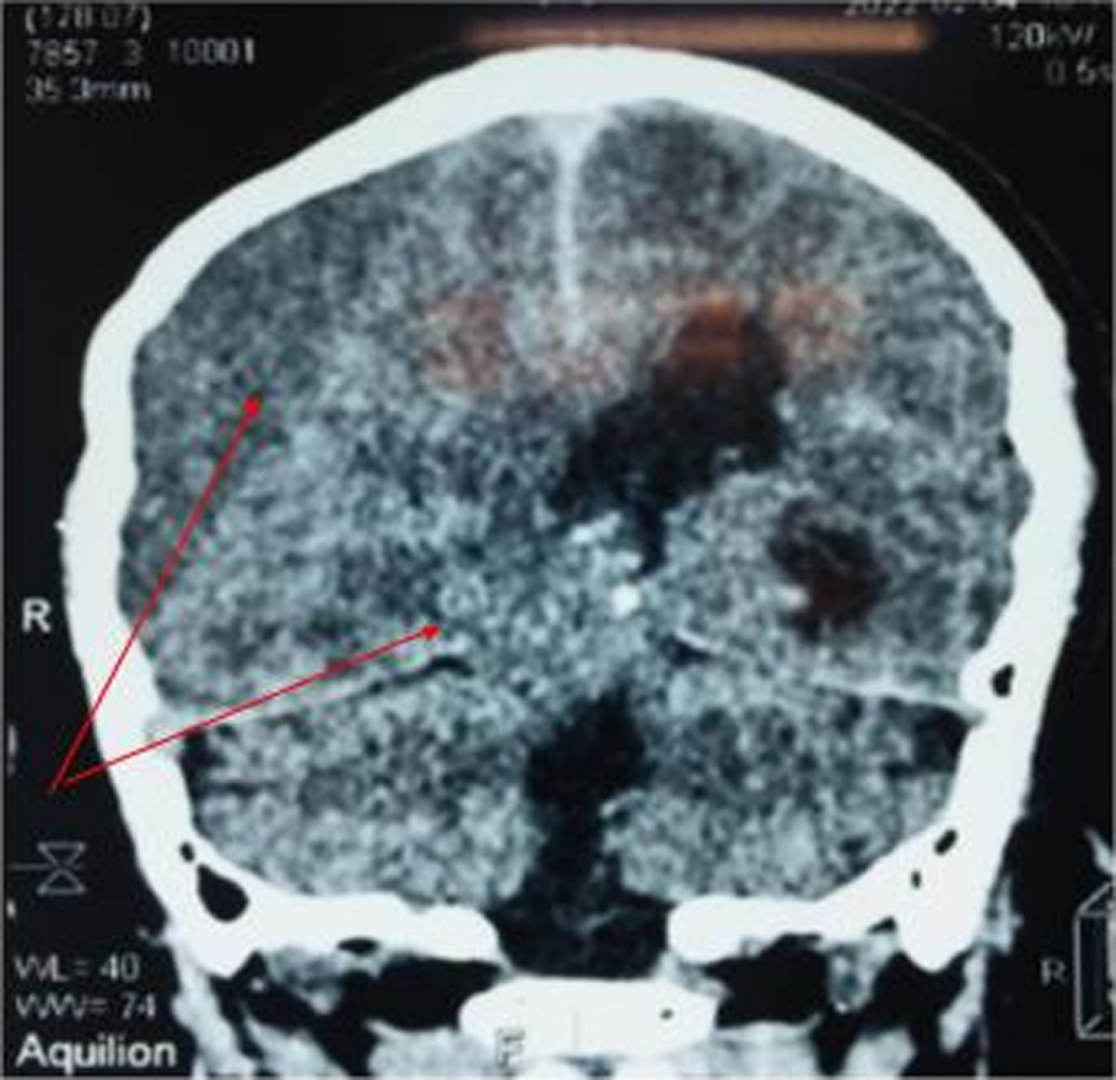
Cerebral CT image, coronal reconstruction showing a large right hemispheric chronic subdural hematoma with a temporal hernia.

A brain scan was performed which showed a large right hemispheric subdural hematoma with subfalcoral involvement (Figure 1) and a Kernohan–Woltman phenomenon (Figure 2). He was taken to the operating room for evacuation of the hematoma; two drill holes were made, which allowed evacuation under pressure of 300 mL of digested blood. A subdural drain ending in the right occipitotemporal was left. On awakening, the patient had a maximum Glasgow, the right central facial palsy and right hemiparesis had disappeared, and the pupils were of good reflection diameter.

On the second day, the drain was removed after bringing back a total of 150 cc of serohematic fluid, and on the fifth day, he was discharged without any neurological deficit.

## 3 DISCUSSION

The corticospinal pathways originate from the motor cortex in the frontal lobes and descend through the internal capsules and then through the midbrain and annular protrusions, before the majority of these motor fibers (80%) undergo decussation in the medulla oblongata.^7^ This decussation of these motor fibers will explain the fact that the left cerebral motor cortex controls the movements of the right side of the human body and vice versa. It is therefore logical that a lesion of a cerebral hemisphere with compression of the motor fibers passing through it should manifest itself by a contralateral motor deficit. However, this clinic is paradoxical in a rare situation.

**FIGURE 3:**
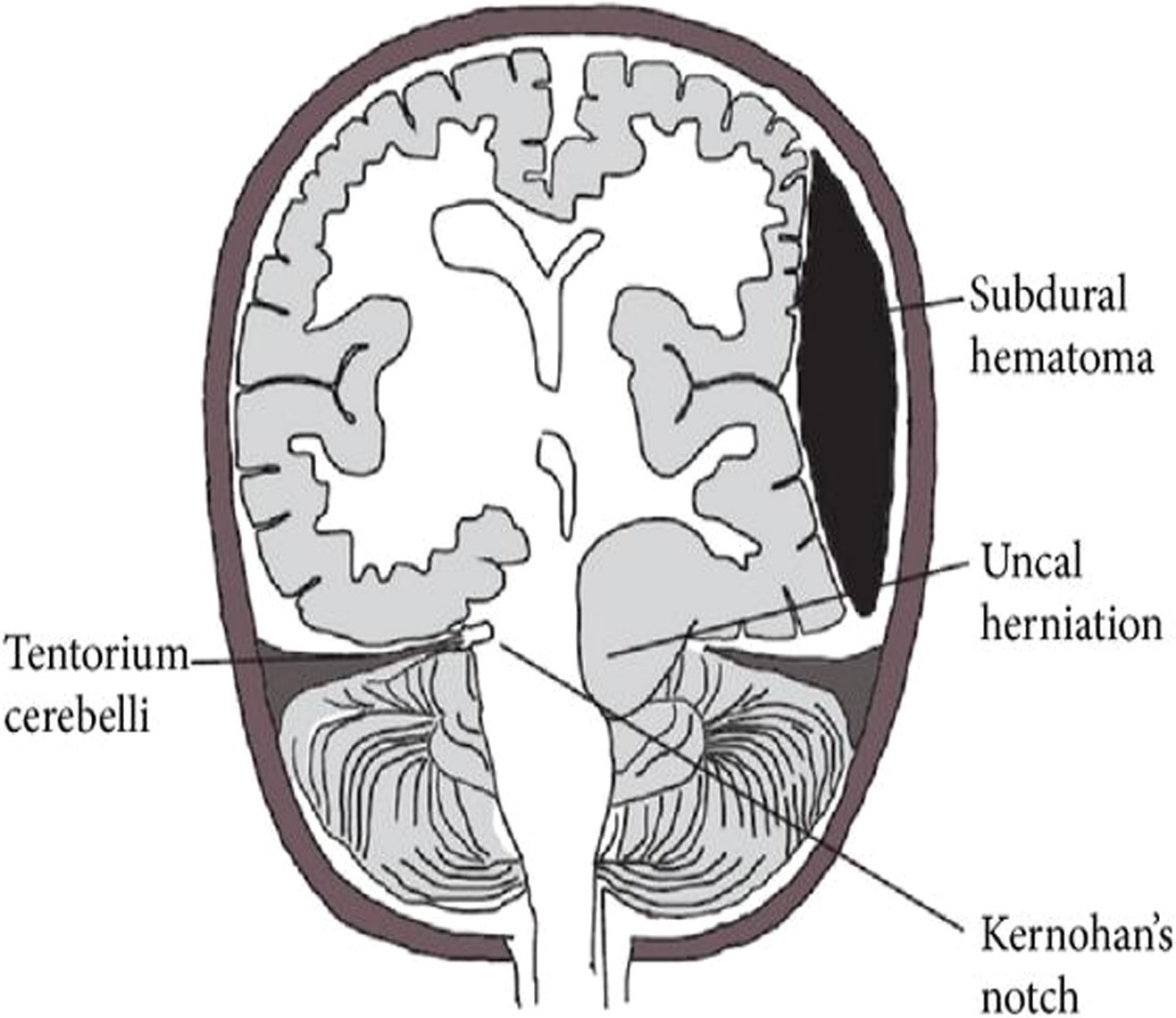
Schematic representation of Kernohan–Woltman notch phenomenon.^5^, ^6^

The Kernohan–Woltman notch phenomenon (Figure 3) is a deceptively localized neurological sign that manifests as a motor deficit ipsilateral to the primary lesion and occurs in the setting of a transtentorial hernia, where the contralateral cerebral peduncle is compressed against the free edge of the tentorium, resulting in compression of fibers of the descending corticospinal tract.^2^

This paradoxical neurologic sign or false localization was first described in 1929 by Kernohan and colleagues during autopsy examination of a patient with a brain tumor whose brain was nicked by the peduncle of a contralateral hernia.^8^ This phenomenon has been associated with brain tumors and supratentorial hematomas.^3^, ^9^

Compression of the contralateral peduncle is thought to disrupt fibers of the corticospinal tract above the decussation, resulting in hemiparesis ipsilateral to the primary lesion.^3^ Our patient presented with a right hemiparesis ipsilateral to a large chronic subdural hematoma responsible for the Kernohan–Woltman phenomenon visible on brain CT.

Other clinical signs, apart from hemiplegia or hemiparesis, may be observed, including ipsilateral mydriasis, divergent gaze, or ptosis secondary to the primary lesion with oculomotor nerve involvement, and ipsilateral paralysis of the superior motor nerve of the face due to involvement of corticobulbar fibers of the cerebral peduncle.^3^, ^10^ And other authors mention altered consciousness as a clinical sign often found.^11^ Our patient presented on admission with altered consciousness; right mydriasis and right central facial palsy ipsilateral to his large chronic subdural hematoma.

A good neurological examination combined with medical imaging plays an important role in the diagnosis. Brain magnetic resonance imaging may show T2 hyperintensity of the contralateral cerebral peduncle.^3^ A brain scan may be performed. Several authors have suggested that some individuals are susceptible to developing this syndrome due to anatomical factors.^12^, ^13^ Adher and Milhorat autopsied and analyzed the tentorium morphology of 100 individuals, and found that they differed in the diameter of the tentorium notch, which ranged from 24.5 to 39.0 mm.^14^ Based on these data, it is possible to speculate that individuals with a smaller diameter of this structure tend to herniate and may generate contralateral compression of the cerebral peduncle more readily than those with a larger diameter.

It is very crucial to recognize this syndrome because of the risk of error in the neurosurgical procedure by operating on the wrong side with respect to the location of the lesion. Wolf RFE and colleagues^15^ reported an unfortunate incident in which a patient who had a subdural hematoma after being hit by a golf ball, the patient had a motor deficit on the left side and the brain scan showed an ipsilateral subdural hematoma. The surgeons thought that the CT indexes were misplaced, so they performed a craniotomy on the side opposite to the motor deficit (the healthy side), which did not reveal a hematoma; a follow‐up brain CT scan was performed postoperatively and showed that the hematoma was contralateral to the surgical site.^15^

## 4 CONCLUSION

The Kernohan–Wotman phenomenon is a rare, paradoxical situation but not to be ignored in clinical neurosurgical practice. Several factors can lead to this phenomenon among which we have cerebral hematoma after cranioencephalic trauma. A good neurological examination and magnetic resonance imaging or CT scan are necessary for diagnosis. This phenomenon should attract the attention of clinicians in order to avoid unfortunate incidents such as craniotomy on the wrong side in neurosurgical practice.

## AUTHOR CONTRIBUTIONS

**Romo Bujiriri Murhega:** Conceptualization; writing – original draft; writing – review and editing. **Maheshe Balemba Ghislain:** Supervision; validation. **Paterne Safari Mudekereza:** Supervision; validation. **Sudi Musilimu:** Writing – original draft. **Igega Bisimwa:** Visualization; writing – original draft. **Paul Munguakonkwa Budema:** Supervision; validation. **Léon‐Emmanuel Mubenga:** Supervision; validation.

## CONFLICT OF INTEREST STATEMENT

The authors have no conflict of interest to declare.

## FUNDING INFORMATION

None.

## ETHICS STATEMENT

This case report received ethical clearance from the Ethical committee of the catholic University of Bukavu.

## PATIENT CONSENT STATEMENT

Written informed consent was signed by the patient prior to the publication of this paper.

## Data Availability

All the materials used in this study are available on request.

## References

[1] Derakhshan I . Letter to the editor: Kernohan's contributions to neurosurgery. Neurosurg Focus. 2014;37(4):E22. doi:10.3171/2014.6.FOCUS14286 25270142

[2] Gimarc K , Massagli TL . Kernohan–Woltman notch phenomenon in two patients with subdural hematoma and ipsilateral hemiparesis. Am J Phys Med Rehabil. 2020;99(12):1195‐1196. doi:10.1097/PHM.0000000000001427 32282365

[3] Zhang CH , DeSouza RM , Kho JSB , Vundavalli S , Critchley G . Kernohan–Woltman notch phenomenon: a review article. Br J Neurosurg. 2017;31(2):159‐166. doi:10.1080/02688697.2016.1211250 27781487

[4] Carrasco‐Moro R , Castro‐Dufourny I , Martínez‐San Millán JS , Cabañes‐Martínez L , Pascual JM . Ipsilateral hemiparesis: the forgotten history of this paradoxical neurological sign. Neurosurg Focus. 2019;47(3):E7. doi:10.3171/2019.6.FOCUS19337 31473680

[5] Pereira C . Kernohan–Woltman notch phenomenon – case report. Arq Bras Neurocir. 2016;5:56. doi:10.1055/s-0036-1593975

[6] Panikkath R , Panikkath D , Lim SY , Nugent K . Kernohan's notch: a forgotten cause of hemiplegia‐CT scans are useful in this diagnosis. Case Report Med. 2013;2013:296874. doi:10.1155/2013/296874 PMC3853108 24348572

[7] Kernohan JW . Incisura of the crus due to contralateral brain tumor. Arch NeurPsych. 1929;21(2):274. doi:10.1001/archneurpsyc.1929.02210200030004

[8] Zafonte RD , Lee CY . Kernohan–Woltman notch phenomenon: an unusual cause of ipsilateral motor deficit. Arch Phys Med Rehabil. 1997;78(5):543‐545. doi:10.1016/s0003-9993(97)90174-x 9161379

[9] Carrasco‐Moro R , Abreu‐Calderón F , de Blas‐Beorlegui G , Pascual JM , Ley‐Urzaiz L . Kernohan–Woltman notch phenomenon. Rev Clin Esp (Barc). 2014;214(8):e97‐e99. doi:10.1016/j.rce.2014.05.004 24930846

[10] McKenna C , Fellus J , Barrett AM . False localizing signs in traumatic brain injury. Brain Inj. 2009;23(7):597‐601. doi:10.1080/02699050902973921 19557561 PMC2938049

[11] Larner AJ . False localising signs. J Neurol Neurosurg Psychiatr. 2003;74(4):415‐418. doi:10.1136/jnnp.74.4.415 PMC1738389 12640051

[12] Moon K‐S , Lee J‐K , Joo S‐P , et al. Kernohan's notch phenomenon in chronic subdural hematoma: MRI findings. J Clin Neurosci. 2007;14(10):989‐992. doi:10.1016/j.jocn.2006.05.016 17823049

[13] Carrasco R , Pascual JM , Navas M , Martínez‐Flórez P , Manzanares‐Soler R , Sola RG . Kernohan–Woltman notch phenomenon caused by an acute subdural hematoma. J Clin Neurosci. 2009;16(12):1628‐1631. doi:10.1016/j.jocn.2009.02.015 19766003

[14] Adler DE , Milhorat TH . The tentorial notch: anatomical variation, morphometric analysis, and classification in 100 human autopsy cases. J Neurosurg. 2002;96(6):1103‐1112. doi:10.3171/jns.2002.96.6.1103 12066913

[15] Wolf RF , ter Weeme CA , Krikke AP . Kernohan's notch and misdiagnosis. Lancet. 1995;345(8944):259‐260. 7823740

